# The dynamics of wild *Vitis* species in response to climate change facilitate the breeding of grapevine and its rootstocks with climate resilience

**DOI:** 10.1093/hr/uhaf104

**Published:** 2025-04-10

**Authors:** Mengyan Zhang, Xiaodong Xu, Tianhao Zhang, Zhenya Liu, Xingyi Wang, Xiaoya Shi, Wenjing Peng, Xu Wang, Zhuyifu Chen, Ruoyan Zhao, Wenrui Wang, Yi Zhang, Zhongxin Jin, Yongfeng Zhou, Zhiyao Ma

**Affiliations:** National Key Laboratory of Tropical Crop Breeding, Shenzhen Branch, Guangdong Laboratory of Lingnan Modern Agriculture, Key Laboratory of Synthetic Biology, Ministry of Agriculture and Rural Affairs, Agricultural Genomics Institute at Shenzhen, Chinese Academy of Agricultural Sciences, Buxin road No. 97, Dapeng district, Shenzhen, China; School of Agriculture and Food Science, University College Dublin, Belfield, Dublin 4, Ireland; National Key Laboratory of Tropical Crop Breeding, Shenzhen Branch, Guangdong Laboratory of Lingnan Modern Agriculture, Key Laboratory of Synthetic Biology, Ministry of Agriculture and Rural Affairs, Agricultural Genomics Institute at Shenzhen, Chinese Academy of Agricultural Sciences, Buxin road No. 97, Dapeng district, Shenzhen, China; National Key Laboratory of Tropical Crop Breeding, Shenzhen Branch, Guangdong Laboratory of Lingnan Modern Agriculture, Key Laboratory of Synthetic Biology, Ministry of Agriculture and Rural Affairs, Agricultural Genomics Institute at Shenzhen, Chinese Academy of Agricultural Sciences, Buxin road No. 97, Dapeng district, Shenzhen, China; National Key Laboratory of Tropical Crop Breeding, Shenzhen Branch, Guangdong Laboratory of Lingnan Modern Agriculture, Key Laboratory of Synthetic Biology, Ministry of Agriculture and Rural Affairs, Agricultural Genomics Institute at Shenzhen, Chinese Academy of Agricultural Sciences, Buxin road No. 97, Dapeng district, Shenzhen, China; National Key Laboratory of Tropical Crop Breeding, Shenzhen Branch, Guangdong Laboratory of Lingnan Modern Agriculture, Key Laboratory of Synthetic Biology, Ministry of Agriculture and Rural Affairs, Agricultural Genomics Institute at Shenzhen, Chinese Academy of Agricultural Sciences, Buxin road No. 97, Dapeng district, Shenzhen, China; College of Forestry, Beijing Forestry University, Qinghua road No. 35, Haidian district, Beijing, China; National Key Laboratory of Tropical Crop Breeding, Shenzhen Branch, Guangdong Laboratory of Lingnan Modern Agriculture, Key Laboratory of Synthetic Biology, Ministry of Agriculture and Rural Affairs, Agricultural Genomics Institute at Shenzhen, Chinese Academy of Agricultural Sciences, Buxin road No. 97, Dapeng district, Shenzhen, China; National Key Laboratory of Tropical Crop Breeding, Shenzhen Branch, Guangdong Laboratory of Lingnan Modern Agriculture, Key Laboratory of Synthetic Biology, Ministry of Agriculture and Rural Affairs, Agricultural Genomics Institute at Shenzhen, Chinese Academy of Agricultural Sciences, Buxin road No. 97, Dapeng district, Shenzhen, China; National Key Laboratory of Tropical Crop Breeding, Shenzhen Branch, Guangdong Laboratory of Lingnan Modern Agriculture, Key Laboratory of Synthetic Biology, Ministry of Agriculture and Rural Affairs, Agricultural Genomics Institute at Shenzhen, Chinese Academy of Agricultural Sciences, Buxin road No. 97, Dapeng district, Shenzhen, China; School of Agriculture and Food Science, University College Dublin, Belfield, Dublin 4, Ireland; National Key Laboratory of Tropical Crop Breeding, Shenzhen Branch, Guangdong Laboratory of Lingnan Modern Agriculture, Key Laboratory of Synthetic Biology, Ministry of Agriculture and Rural Affairs, Agricultural Genomics Institute at Shenzhen, Chinese Academy of Agricultural Sciences, Buxin road No. 97, Dapeng district, Shenzhen, China; School of Agriculture and Food Science, University College Dublin, Belfield, Dublin 4, Ireland; National Key Laboratory of Tropical Crop Breeding, Shenzhen Branch, Guangdong Laboratory of Lingnan Modern Agriculture, Key Laboratory of Synthetic Biology, Ministry of Agriculture and Rural Affairs, Agricultural Genomics Institute at Shenzhen, Chinese Academy of Agricultural Sciences, Buxin road No. 97, Dapeng district, Shenzhen, China; National Key Laboratory of Tropical Crop Breeding, Shenzhen Branch, Guangdong Laboratory of Lingnan Modern Agriculture, Key Laboratory of Synthetic Biology, Ministry of Agriculture and Rural Affairs, Agricultural Genomics Institute at Shenzhen, Chinese Academy of Agricultural Sciences, Buxin road No. 97, Dapeng district, Shenzhen, China; National Key Laboratory of Tropical Crop Breeding, Shenzhen Branch, Guangdong Laboratory of Lingnan Modern Agriculture, Key Laboratory of Synthetic Biology, Ministry of Agriculture and Rural Affairs, Agricultural Genomics Institute at Shenzhen, Chinese Academy of Agricultural Sciences, Buxin road No. 97, Dapeng district, Shenzhen, China; National Key Laboratory of Tropical Crop Breeding, Shenzhen Branch, Guangdong Laboratory of Lingnan Modern Agriculture, Key Laboratory of Synthetic Biology, Ministry of Agriculture and Rural Affairs, Agricultural Genomics Institute at Shenzhen, Chinese Academy of Agricultural Sciences, Buxin road No. 97, Dapeng district, Shenzhen, China; National Key Laboratory of Tropical Crop Breeding, Shenzhen Branch, Guangdong Laboratory of Lingnan Modern Agriculture, Key Laboratory of Synthetic Biology, Ministry of Agriculture and Rural Affairs, Agricultural Genomics Institute at Shenzhen, Chinese Academy of Agricultural Sciences, Buxin road No. 97, Dapeng district, Shenzhen, China; National Key Laboratory of Tropical Crop Breeding, Tropical Crops Genetic Resources Institute, Chinese Academy of Tropical Agricultural Sciences, Xueyuan road No. 4, Longhua district, Haikou, China; National Key Laboratory of Tropical Crop Breeding, Shenzhen Branch, Guangdong Laboratory of Lingnan Modern Agriculture, Key Laboratory of Synthetic Biology, Ministry of Agriculture and Rural Affairs, Agricultural Genomics Institute at Shenzhen, Chinese Academy of Agricultural Sciences, Buxin road No. 97, Dapeng district, Shenzhen, China

## Abstract

Climate change presents significant challenges to agricultural suitability and food security, largely due to the limited adaptability of domesticated crops. However, crop wild relatives maintain greater diversity and are well adapted to various environments. This study evaluates the potential distributional responses of grapevine (*Vitis vinifera* L.) and its wild relatives (*Vitis* spp.) to future climate change using the maximum entropy model. We reveal that the annual mean temperature is the primary factor determining the potential distribution of cultivated grapes. By 2080, under the SSP585 scenario, suitable areas for wine and table grapes are predicted to decline by 1.5 million and 1.3 million km ^2^, respectively. The results suggest that grape cultivation, especially for table grapes, is highly vulnerable to future climate change. In contrast, approximately 70% of wild grapes are projected to demonstrate robust adaptability to future conditions. For example, wild grapes from North America, such as *Vitis rotundifolia* and *Vitis labrusca*, and from East Asia, such as *Vitis heyneana* and *Vitis davidii*, are projected to demonstrate significant adaptability in response to future climate change. These wild grapes are valuable genetic resources for improving the resilience of cultivated grapes through rootstock development and breeding programs to face the climate change. Our results predict the potential future distribution areas of wild grapes and highlight the critical role of their genetic resources in grape breeding for promoting adaptation to climate change.

## Introduction

Global climate change has significant implications for food security and agricultural sustainability, affecting crop growth and overall productivity [1, 2–4]. Climate change may reduce the acreage of important crops, as rising temperatures and sea levels increasingly challenge their ability to thrive in traditional growing regions [5, 6]. For example, wheat is considered the most vulnerable crop in eastern Africa, with projections indicating a yield reduction of up to 72% by the end of this century [7]. This issue extends beyond wheat, as the productivity of grain legumes will also be affected by the shrinking and degradation of arable land due to global climate change [8]. Additionally, climate change is predicted to have adverse effects on major crops, particularly in tropical regions like Brazil, where maize production is experiencing significant declines due to delayed rainy seasons and rising temperatures. This highlights the urgent need for adaptive agricultural strategies to ensure food security [9, 10]. Overall, these challenges underscore the importance of resilient agricultural practices to mitigate the effects of climate change on our crop production.

The crop wild relatives (CWRs), compared to domesticated cultivars, have been shown to be more reliant to global climate change and could adapt to harsh environments [11]. Increasing agricultural plant diversity (agrobiodiversity) and the utilization of CWRs are crucial for enhancing crop adaptability to future climate challenges [12]. Compared to domesticated cultivars, CWRs possess untapped genetic diversity and provide valuable traits, such as disease resistance and drought tolerance, which can be effectively utilized for crop improvement with genome editing techniques in response to changing climatic conditions [13–15]. Increasing attention has focused on the role of CWRs in promoting climate adaptation of crops recently. It has been suggested that crop sustainability and resilience in sub-Saharan Africa can be enhanced through the use of CWRs for crops such as potato, squash, and finger millet [2, 16]. CWRs play a key role in assessing the impacts of climate change on crops and in developing adaptation strategies. Similarly, the global distribution and conservation status of rice wild relatives have been evaluated, with strategies to ensure the resilience of rice genetic resources in response to climate change [17]. Concurrently, the investigation of alternative varieties, rootstocks, or geographic relocations may be explored [18, 19]. In summary, the impact of global climate change on crops highlights the importance of using CWRs in agricultural strategies to enhance crop resilience.

Grapevine (*Vitis vinifera* L.) is one of the most important fruit crops globally, widely cultivated for both fresh consumption and wine production [3, 20–24, 72]. However, viticulture is highly sensitive to climate change, which significantly limits its suitable growing regions, quality, and productivity [25, 26]. Climate change affects grapevine growth by influencing phenological stages such as bud burst, flowering, and ripening [27]. Changes in temperature and precipitation can alter the timing of these stages, potentially impacting grapevine distribution and productivity [18, 28]. Furthermore, climate change can also impact grape quality by influencing sugar accumulation, acidity levels, and phenolic compounds [29, 30]. Higher temperatures during the growing season, as projected under future climate scenarios, may increase sugar accumulation, affecting the flavor profile and overall quality of the grapes. Additionally, climate change might have a detrimental impact on grapevines by increasing pest and disease pressure and decreasing crop yield [31]. Wild grape relatives in the *Vitis* genus are important genetic resources for developing rootstocks and breeding with desirable traits, including resistance to biotic and abiotic stresses, such as Pierce’s disease, cold, drought, and salinity [32, 33, 73, 75]. For example, studies have found that rootstocks can enhance the tolerance of plants to abiotic stresses (such as drought and salt stress) by restricting the transport of heavy metal ions and through detoxification processes in the root system [34]. Additionally, the genetic background and root traits of rootstocks can also influence the growth and quality of grapes. Certain wild grape species, such as *Vitis berlandieri*, exhibit high heritability and genetic variation in root traits, which can be transferred to cultivated varieties through grafting, thereby improving their adaptability to climate change [35]. Although the influence of environmental niches on the distribution and growth of wild grapes has attracted researchers’ attention [36–40], the assessment of the potential of wild grape relatives to respond to future climate is still lacking.

In this study, we selected two scenarios: SSP245, characterized by moderate greenhouse gas emissions and relatively manageable climate change impacts, and SSP585, which features higher greenhouse gas emissions and more pronounced climate change impacts. These scenarios provide a comprehensive coverage of the potential range of future climate change, offering a more representative and forward-looking foundation for our analysis. We employed the maxent model based on the maximum entropy principle, leveraging its strengths in processing environmental variables and predicting species distribution. We made detailed estimates of the distribution dynamics of cultivated grapes and their wild relatives (*Vitis* L.) in response to future climate change. This method not only accurately captures changes in environmental variables but also provides a strong scientific basis for developing adaptive strategies in grape cultivation. We aim to simulate the niche distribution area and investigate climate factors preferment of cultivated grape and its wild relatives, evaluate the response of cultivated grape and its wild relatives to future climate change, and identify wild species with potential for breeding and as rootstocks in response to future climate change.

## Results

### Potential distribution of cultivated grapes (*V. vinifera* ssp. *vinifera*) in response to future climate change

**Figure 1:**
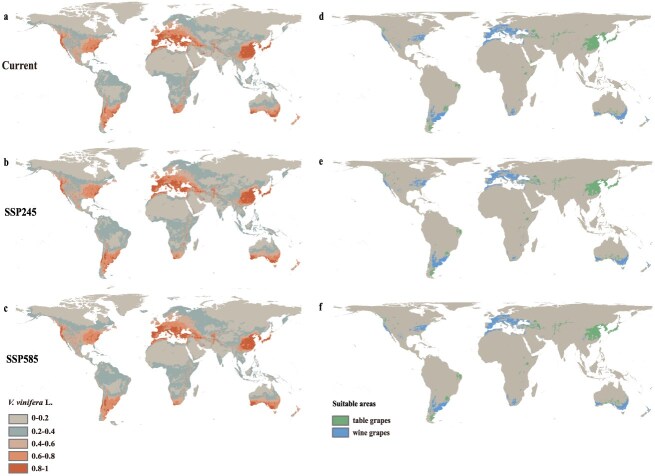
Suitable distribution regions of cultivated grapes (*V. vinifera*) predicted with maximum entropy model under different climate change scenarios (a, d) present, (b, e) under the SSP245 scenario during 2081–2100, and (c, f) under the SSP585 scenario during 2081–2100. The left panel was for all the domesticated grapes, and the right panel represent estimation for table grapes (green) and wine grapes (blue), respectively.

**Figure 2:**
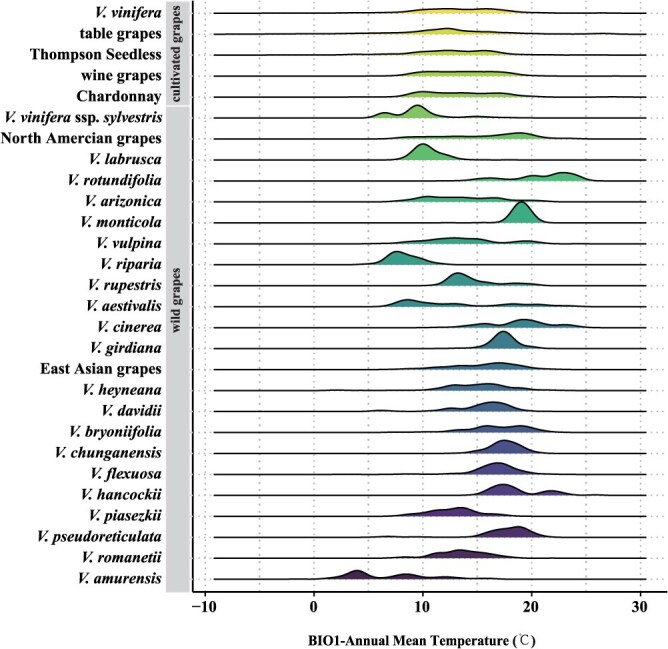
Annual Mean Temperature (bio1) tolerance of cultivated grapes and wild grapes. The *x*-axis distribution indicates plasticity in response to different annual mean temperatures. Each row on the *y*-axis distribution represents the names of cultivated grapes and wild grapes. The height of each peak represents the assigned probability.

Understanding the relationship between species distribution and environmental factors is crucial for evaluating the impact of climate change on plant populations. Under current climate conditions, the suitable distribution areas for cultivated grapes (*V. vinifera*) are widely spread across East Asia, Europe, North America, and southern Australia (Fig. 1a), with distinct distribution patterns for table grapes and wine grapes (Fig. 1f). And under the SSP245 (Intermediate Development) and SSP585 (Fossil-Fueled Development) scenarios, two of the Shared Socioeconomic Pathways (SSPs) considered, the potential future trajectories of greenhouse gas concentrations are evaluated. SSP245 represents an intermediate development path, while SSP585 reflects a high dependence on fossil fuels. Under future climate condition, the suitable distribution for *V. vinifera* is expected to be significantly reduced (Fig. 1). This suggests that high greenhouse gas emissions could potentially have a substantial impact on the geographical distribution of *V. vinifera*, indicating that extreme temperatures may significantly negatively affect the spatial distribution of grapes. These results emphasize the critical role of temperature in determining the distribution of *V. vinifera* and also point to the challenges it may encounter due to climate change (Fig. 1). Using the maxent species distribution model, among the 19 climate factors, the Annual Mean Temperature (bio1) was identified as the primary factor influencing the potential distribution of *V. vinifera* (Fig. S1), which is followed by the Mean Temperature of the Coldest Quarter (bio11) and the Minimum Temperature of the Coldest Month (bio6). The Annual Mean Temperature (bio1) has been identified as a primary factor influencing the potential distribution of grapes. To assess grape growth suitability under different temperatures, we analyze the impact of bio1 on both wild and cultivated grapes. The analysis indicates that these grape varieties show different temperature adaptability, with bio1 having a greater impact on cultivated grapes (Fig. S1). Wild grapes exhibit greater temperature adaptability than cultivated grapes, allowing them to thrive in diverse climatic conditions. Among them, *Vitis cinerea*, *Vitis rotundifolia*, and *Vitis hancockii* show remarkable tolerance to high temperatures. While *V. vinifera* ssp. *sylvestris*, *Vitis amurensis*, and *Vitis aestivalis* varieties exhibit strong resistance to low temperatures, *V. amurensis* is particularly notable for its exceptional cold tolerance (Fig. 2). It is worth noting that *V. vinifera* ssp. *sylvestris* and *V. amurensis* were affected by the Quaternary glaciation, resulting in geographical isolation or refuge groups within the species, which led to certain differences in the response of the groups to climate factors. We further conducted prediction analyses of different geographical distribution groups within these two species. Under the SSP245 and SSP585 scenarios, with the intensification of extreme weather events, the suitable areas of *V. vinifera* ssp. *sylvestris* (Fig. S2a–d) and *V. amurensis* (Fig. S2e–h) showed a trend of gradual reduction. Under the SSP585 scenario, the reduction trend of *V. amurensis* (Fig. S2f and h) was more obvious. In addition to temperature, precipitation is also an important factor affecting grape growth, especially in the Precipitation of Warmest Quarter (bio18) and Precipitation of Coldest Quarter (bio19). Notably, the growth and distribution of European (*V. vinifera* ssp. *sylvestris*) and North American wild grapes are significantly affected by the changes in precipitation in a specific season (Fig. S3). These results emphasize the decisive influence of temperature and precipitation on the growth and distribution of grapes. They not only highlight the key role of temperature in determining grape distribution but also reveal the challenges grapes may face due to climate change. These findings are crucial for understanding the response of grapes to climate change and developing adaptive management strategies.

Under SSP585 scenario, projections indicate that the current suitable regions for *V. vinifera* will persist, encompassing an area of around 125.4 × 10^5^ km^2^ (Table S1). The suitable areas for wine grapes and table grapes are expected to increase to 13.36 × 10^5^ and 19.12 × 10^5^ km^2^ (Table S1). Despite overall expansion in the areas suitable for viticulture, the potential suitable areas for wine grapes decreased to 15.04 × 10^5^ km^2^, and for table grapes, it decreased to 13.26 × 10^5^ km^2^ (Table S1). The greater reduction for wine grapes compared to table grapes indicates that wine grapes may be more sensitive to climate change, or their suitable growing conditions could be more negatively impacted.

**Figure 3:**
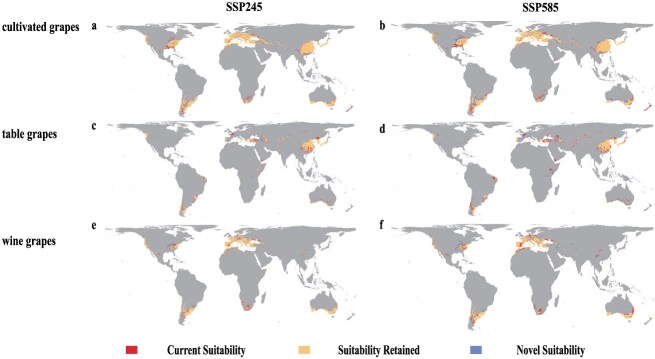
Suitable distribution regions for cultivated grapes (*V. vinifera*), table grapes, and wine grapes estimated using maximum entropy model; (a, b) cultivated grapes (*V. vinifera*), (c, d) table grapes, and (e, f) wine grapes, with future projections for the years 2081–2100 under the SSP245 and SSP585 climatic scenarios. The projected bioclimatic suitability for Thompson Seedless and Chardonnay under the SSP245 and SSP585 scenarios can be found in the Supplementary Material (Fig. S4).

In detail, most of the current suitable distribution region is projected to be retained (Fig. 3c and d) in larger areas of current table grapes-producing regions, especially in China; however, the model predicts a decline in suitability in the southern regions. Meanwhile, the suitability of Chile, the main table grapes producer in South America, is also declining (Fig. 3c and d), whereas the regions of northern China and northern Turkey are projected to become suitable in the future (Fig. 3c and d). Specifically, current suitability is projected to be retained (Fig. 3e and f and Fig. S4c and d) in many traditional wine-producing regions (such as France and Italy), under SSP245 and SSP585 scenarios. Suitability is projected to decline (Fig. 3e and f and Fig. S4c and d) in Spanish regions and northeastern Australia (Queensland). Conversely, northern North American and two important non-Mediterranean wine-producing regions (non-Mediterranean Australia and New Zealand) are projected to expand in the future (Fig. 3e and f). As for Thompson Seedless, the most widely distributed table grapes, there is an observed trend of contraction in its suitable distribution range in southern China and Europe, as indicated by the red regions in Fig. S4a and b; conversely, in North America, the suitable areas for Thompson Seedless are expanding, as shown by the blue regions in the same figures. Chardonnay, the most widely distributed wine grapes, follows the same trend as wine grapes in terms of climate preferences. Interestingly, our analysis reveals that the retained suitability areas for wild grapes are comparable to 63% of those for cultivated grapes in the SSP245 and 65% in the SSP585 scenarios, respectively. This suggests that some areas currently suitable for grape cultivation may become unsuitable in the future.

### Grape wild relatives in response to climate change

**Figure 4:**
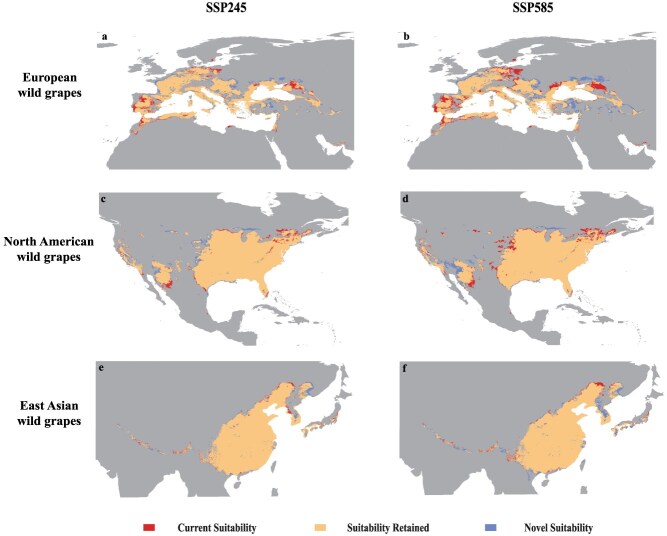
Maxent model projected bioclimatic suitability for European wild grapes (*V. vinifera* ssp. *sylvestris*), North American wild grapes, and East Asian wild grapes; (a, b) European wild grapes (*V. vinifera* ssp. *sylvestris*), (c, d) North American wild grapes, and (e, f) East Asian wild grapes, with future projections for the years 2081–2100 under the SSP245 and SSP585 climatic scenarios.

We compared the responses of European (*V. vinifera* ssp*. sylvestris*), North American, and East Asian wild grapes species to climate change to identify those that could potentially improve the adaptability and resistance of domesticated grapes. The results show that the current suitable areas are expected to be retained (Fig. 4a–f) in larger areas of the three wild grape regions. Among them, North American wild grapes have the largest area of 30.37 × 10^5^ km^2^, followed by East Asian wild grapes and *V. vinifera* ssp. *sylvestris*, with 27.05 × 10^5^ and 23.88 × 10^5^ km^2^, respectively (Table S1). For *V. vinifera* ssp. *sylvestris*, suitability is declining in the southeastern Europe, with a total decrease reaching 5.13 × 10^5^ km^2^ (Table S1). In terms of the expansion–contraction ratio, under the SSP245 scenario, European wild grapes (*V. vinifera* ssp. *sylvestris*) exhibited a significant expansion trend in the future, increasing by 3.6%, with East Asian wild grapes following at 0.53%, while the suitability for North American wild grapes decreased by 0.32% (Fig. S5). As the climate gradually worsens, under the SSP585 scenario, the *V. vinifera* ssp. *sylvestris* expansion–contraction ratio decreases to 1.8%. On the contrary, the East Asian wild grapes and North American wild grapes increase significantly to 4.19% and 3.38%, respectively (Fig. S5). This suggests that the East Asian and North American wild grapes demonstrate a greater capacity to adapt to future adverse weather conditions compared to *V. vinifera* ssp. *sylvestris.*

### Future suitability of North American wild grapes

**Figure 5:**
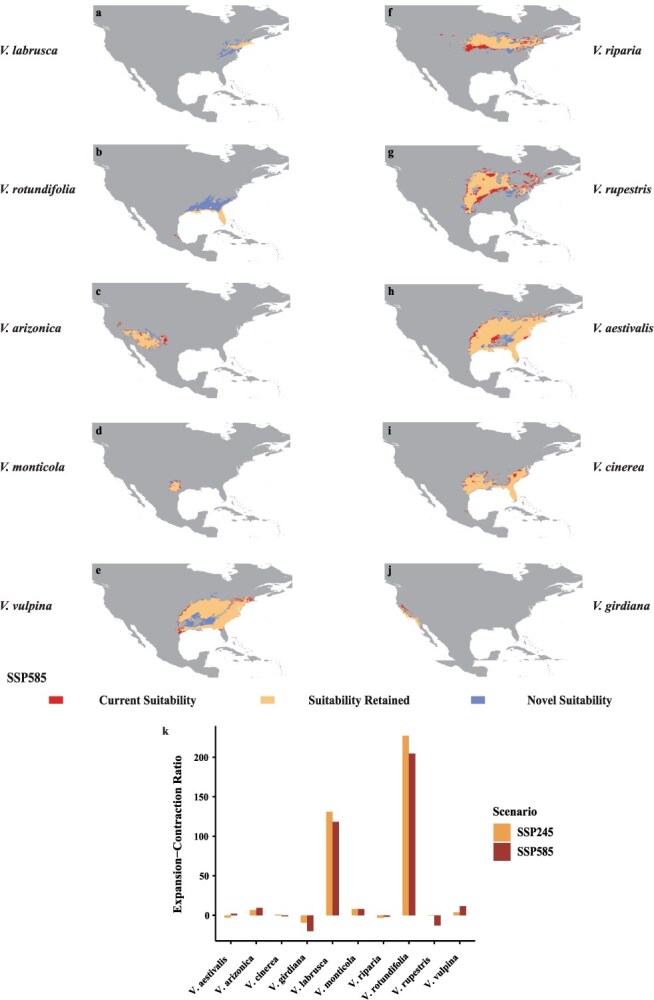
Maxent model projected bioclimatic suitability for North American wild grapes (a) *V. labrusca*, (b) *V. rotundifolia*, (c) *V. arizonica*, (d) *V. monticola*, (e) *V. vulpina*, (f) *V. riparia*, (g) *V. rupestris*, (h) *V. aestivalis*, (i) *V. cinerea*, (j) *V. girdiana*, with future projections for the years 2081–2100 under the SSP585 climatic scenarios; future projections under the SSP245 climate scenario are in the Supplementary Material (Fig. S6). (k) Net suitability changes for the distribution of North American wild grapes. Bar plots show expansion–contraction ratio of change in area suitable for grape-growing regions projected by maxent model for SSP245 (yellow) and SSP585 (red) scenarios.

An analysis of distribution of 10 North American wild grape species found that *V. rotundifolia* is expected to experience the largest expansion of suitable areas in the future (Fig. 5b), especially in the southeastern regions of North American. To better understand these geographic shifts, we examine expansion–contraction ratio analysis. In North American grapes, the ensemble mean increases in suitable areas are 227% under SSP245 scenario and 205% under SSP585 scenario (Fig. 5k). Followed by *Vitis labrusca* (Fig. 5a), large newly suitable areas are projected in regions of North America, accounting for 131% under SSP245 scenario and 118% under SSP585 scenario, respectively (Fig. 5k). They may be able to adapt to the warmer climate and changing patterns of precipitation. Meanwhile, the cultivation areas of *Vitis vulpina*, *V. aestivalis*, *Vitis monticola*, and *Vitis arizonica* have also shown an expanding trend, with respective increases of 11.49%, 9.28%, 7.88%, and 2.01%, while the distribution areas of *Vitis rupestris*, *Vitis riparia*, and *V. cinerea* are facing the risk of contraction. Under the SSP585 scenario, the mean suitability decline rates are 12.91%, 1.91%, and 1.37%, respectively, mainly in the middle and lower parts of North America (Fig. 5f, g, i). Unfortunately, as climate conditions worsen, the environmental requirements for *Vitis girdiana* can no longer be met in North America, indicating that *V. girdiana* has become unsuitable for distribution there (Fig. 5j).

### Future suitability of East Asian wild grapes

**Figure 6:**
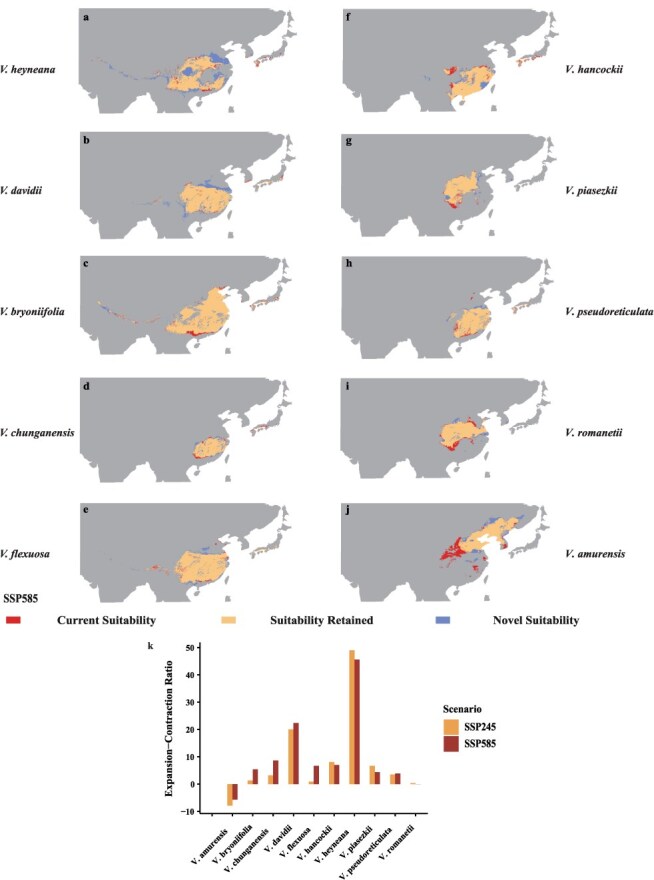
Maxent model projected bioclimatic suitability for East Asian wild grapes (a) *V. heyneana*, (b) *V. davidii*, (c) *V. bryoniifolia*, (d) *V. chunganensis*, (e) *V. flexuosa*, (f) *V. hancockii*, (g) *V. piasezkii*, (h) *V. pseudoreticulata*, (i) *V. romanetii*, (j) *V. amurensis*, with future projections for the years 2081–2100 under the SSP585 climatic scenarios; future projections under the SSP245 climate scenario are in the Supplementary Material (Fig. S7). (k) Net suitability change for the distribution of East Asian wild grapes. Bar plots show expansion–contraction ratio of change in area suitable for grape-growing regions projected by maxent model for SSP245 (yellow) and SSP585 (red) scenarios.

For East Asian wild grapes, we analyzed the sensitivity and adaptability of 10 grape species to climate change. Among them, *Vitis heyneana* shows an ensemble mean increase in suitable areas of 49% in China under SSP245 and 46% in SSP585 (Fig. 6k). Large increases in ecological footprint are projected in eastern, central, and western China (Fig. 6a, marked in blue). In contrast, *Vitis davidii* displays an opposite trend: under SSP585 scenario, suitable areas increase by 22%, compared to a 2.3% increase under SSP245 scenario. *Vitis bryoniifolia*, *Vitis chunganensis*, *Vitis flexuosa*, *V. hancockii*, *Vitis piasezkii*, and *Vitis pseudoreticulata* also show positive expansion trends (Fig. 6k), with projected expansions ranging from 0.89% to 8.11% under the SSP245 scenario and from 3.89% to 8.65% under the SSP585 scenario, attributed to their adaptability to environmental changes. In contrast, the suitable areas for *V. amurensis* are anticipated to contract. Under the SSP245 scenario, the suitable areas are projected to decrease by 7.91%, while under the SSP585 scenario, a reduction of 5.66% is expected (Fig. 6k), mainly concentrated in the middle reaches and middle and lower reaches of the Yellow River in China (Fig. 6j). Unlike other species, *Vitis romanetii* has an expansion–contraction ratio of zero for its suitable areas, indicating it is at a critical threshold. Further research and adaptive farming strategies may be necessary to ensure its viability. In general, under the impending climate change, 8 of the 10 species exhibit significant adaptive capabilities. These species stand out for future agricultural efforts due to their positive growth trajectories and resilience to anticipated climate changes. Their favorable rate of expansion contrasts with that of *V. amurensis*, which is facing contraction. This emphasizes their inherent adaptability and potential to thrive in the face of a changing climate.

## Discussion

### The response of grapevine and its wild relatives to climate change

The decline in crop suitability caused by global climate change may result in significant economic and conservation impacts. The extent of these impacts varies depending on crop type, geographic region, and adaptation strategies, highlighting the urgent need for targeted agricultural interventions to enhance resilience and ensure global food security [41–46]. Hannah *et al.* [25] discussed the potential impact of climate change on the expansion of grape-growing areas to higher latitudes and altitudes, particularly in the northern regions of North America and Europe. While these areas are currently unsuitable for viticulture, they are predicted to become viable for grape cultivation in the future. These regions are recognized as emerging cold-climate grape-growing areas. In addition to anthropogenic activities (such as native vegetation removal, deep tillage, soil sterilization, and the application of fertilizers and pesticides), the impact on soil type and structure plays a crucial role in determining the key conditions for grape growth. Furthermore, climate change, characterized by rising temperatures and changing precipitation patterns, presents a significant threat to the global wine industry [47, 48, 76]. However, a comprehensive evaluation of the impacts of future climate change on cultivated grape and its wild relative remains necessary. Our results further underscore the profound implications of climate change on viticulture and suggest that the potential range expansion of wild grapes in North America as climate conditions evolve presents both opportunities and challenges (Fig. 5). In contrast, under the SSP585 scenario, which projects a significant increase in greenhouse gas emissions, we predict a substantial reduction in the areas suitable for wine grape cultivation. This change could make some traditional grape cultivation regions unsuitable for existing grape varieties, potentially leading to losses of 15.04 × 10^5^ km^2^ for wine grapes and 13.26 × 10^5^ km^2^ for table grapes (Table S1). This change may render traditional grape-growing regions unsuitable for current varieties, requiring the development of new varieties that can adapt to climate change. Furthermore, our analysis indicates that the expansion of suitable areas for table grapes exceeds that of wine grapes, suggesting that table grapes may be more resilient to the severe weather conditions expected in the future (Fig. 3 and Table S1). This unexpected finding suggests that table grapes have a greater potential to thrive under projected environmental stresses compared to wine grapes in the context of climate change. Compared to cultivated grapes, wild grapes may have a greater ability to adapt to future climate change. North American and East Asian wild grapes, in particular, may exhibit even greater adaptability than European ones, such as *V. vinifera* ssp. *sylvestris* (Fig. 4). For instance, wild grapes in North America and East Asia have demonstrated remarkable adaptability to extreme temperature changes (Figs 5k and 6k). Their ability to survive and thrive in warmer climates can be attributed to the genetic diversity shaped by long-term natural selection (Figs 5 and 6). The genetic diversity and stress tolerance traits in these wild grapes could serve as invaluable resources for breeding programs aimed at developing new cultivars better able to withstand the environmental pressures of climate change. As global climate change progresses, this insight could have a significantly impact on the grape industry, potentially leading to a repositioning of table and wild grapes to ensure economic viability and the ability of agriculture against future climate uncertainties. The urgency of adapting agricultural practices and the need for innovation in grape breeding highlight the critical need to ensure the sustainability and evolution of the viticulture industry in the face of global warming.

In response to climate challenges, strategic crop switching and relocation in the United States may reduce the decline in agricultural profits by up to 50% under the SSP585 scenario [49]. Crop migration is a crucial climate adaptation strategy that can protect yields of major crops, although its success depends on overcoming socioeconomic and environmental obstacles [50]. Globally, optimizing cropland distribution through modeling can reduce the environmental impact of agriculture while maintaining food production. Innovative adaptation strategies, such as Ethiopian farmers transitioning to drought-resistant perennial crops like Enset (*Ensete ventricosum*), are enhancing food security in drought-prone regions [51, 52]. Actually, these strategies emphasize the importance of proactive and innovative measures in agriculture to address the complex climate change challenges and ensure the sustainability of both agricultural profits and environmental health. However, these studies also highlight the limitations and challenges of these strategies, including the need for further adaptation measures, uncertainty about long-term sustainability, and potential environmental costs associated with such large-scale shifts in agricultural practices. In summary, while crop switching and migration present viable short- and medium-term solutions to climate change pressures on agriculture, they also emphasize the need for a more comprehensive and sustainable approach to agricultural adaptation that addresses economic, environmental, and regionalcomplexities.

### Wild *Vitis* genetic resources for breeding grapes and its rootstocks with climate resilience

To address climate warming, researchers have found that measures such as increasing irrigation and reducing grape temperatures through misting or sprinkling may be necessary to mitigate the impact of heat stress on grape quality. These measures can be considered part of a long-term adaptation strategy [25]. However, more importantly, researchers have identified valuable genetic resources of CWRs used as rootstocks to enhance the resilience and adaptability of domesticated crops [18]. CWRs are increasingly recognized as valuable genetic resources that enhance crop resilience to various stresses and contribute to agricultural sustainability. Recent advances in genomics and high-throughput techniques are accelerating the development of climate-resilient crops with enhanced stress tolerance and nutritional value [16]. This involves harnessing the genetic potential of CWRs to develop new varieties and polyploids, which is essential for adapting to future climatic conditions. The challenges of integrating CWRs into grape breeding programs include ensuring hybrid compatibility, assessing genetic diversity, managing legal restrictions, and mitigating the risks of disease transmission. However, potential opportunities lie in enhancing crop adaptability and developing new varieties. Solutions may involve marker-assisted breeding and effective risk management strategies. The timeline for CWR integration can vary depending on the region, resources, and research progress. Generally, it may take 5 to 10 years, or even longer, from preliminary research to the development of new varieties, with potential challenges including high costs and uncertainties in market acceptance [53, 54]. In summary, integrating CWRs into crop improvement programs, supported by genomics and conservation strategies, is crucial for building resilience to climate change. This approach not only enhances the genetic base of crops but also ensures the long-term sustainability of global food systems, safeguarding nutritious food for future generations [55, 56]. Our research provides a comprehensive analysis of the adaptability of various wild grape species, including European *V. vinifera* ssp*. sylvestris*, and identifies specific species of East Asian and North American wild grapes that exhibit exceptional abilities to adapt to changing climate patterns. The distribution patterns of *V. vinifera* ssp. *sylvestris* differ between eastern and western populations; however, their predicted distributions align with the overall pattern when merged (Fig. S2b and d and Fig. 4b). Similarly, the growing regions of *V. amurensis* follow a comparable trend (Fig. S2f and h and Fig. 6j). The analysis results of different geographical groups of these two species indicate that different geographical groups do respond differently to environmental changes (Fig. S2). These findings highlight their potential as a genetic bridge for developing more resilient and adaptable grape cultivars. Wild grapes in North America, particularly *V. rotundifolia* and *V. labrusca*, are predicted to undergo substantial increases in suitable distribution areas under different climate change scenarios. *V. rotundifolia* is expected to lead this expansion, with an estimated increase of 227% under SSP245 scenario and 205% under SSP585 scenario (Fig. 5k). In addition to the well-known *V. labrusca* and *V. rotundifolia*, other species such as *V. arizonica*, *V. monticola*, and *V. vulpina* have also demonstrated exceptional adaptability. Similarly, East Asian wild grapes such as *V. heyneana* are predicted to expand their suitable range in the future. Notably, *V. davidii* shows a unique response to climate scenarios, with the expansion–contraction ratio of its suitable habitat under the SSP585 scenario exceeding that projected under the SSP245 scenario (Fig. 6k). Furthermore, other species including *V. bryoniifolia*, *V. chunganensis*, *V. flexuosa*, *V. hancockii*, *V. piasezkii*, and *V. pseudoreticulata* have demonstrated strong adaptability, making them promising candidates for future distribution (Fig. 6k). Previous studies have examined these species, including six wild grapes from the southwestern United States, in comparison with a Chinese wild grape *V. davidii*. Findings suggest that these species have evolved genetic networks and disease resistance genes that enhance their ability to adapt to environmental stresses, such as high temperatures or drought [32, 57].

Additionally, many commercial grape rootstocks (such as *V. riparia* × *V. berlandieri* and *V. rupestris* × *V. berlandieri*) and hybrid varieties are derived from multiple wild species, and their genetic composition and adaptability significantly influence the stress resistance of grape plants [35, 58 ,59]. However, the adaptability of rootstocks may alleviate the climatic stress on primary grape cultivars to a certain extent, which may lead to deviations in model predictions. Therefore, future research should integrate data on rootstock distribution and adaptability to enhance the practical applicability of models. By conducting in-depth analyses of the genetic composition and functions of rootstocks, we can better understand their role in viticulture and develop more precise strategies to address climate change [60]. Overall, these wild grape species demonstrate genetic resilience and potential resistance to environmental stresses, such as drought and heat, which are critical for ensuring the long-term sustainability of grape horticulture. Furthermore, these species have potential in developing rootstocks that can enhance the adaptability of cultivated grapes and help produce high-quality grapes under changing climatic conditions. These findings offer novel insights into grape–environment interactions, ultimately supporting the selection of suitable rootstocks for future viticulture.

### Strategies and recommendations for the grape industry to cope with climate change

The study has conducted an in-depth analysis of the impact of climate change on grape-growing regions and proposed targeted measures to provide scientific guidance for the sustainable development of the grape industry. It is recommended to use greenhouse and field experiments to evaluate the heat tolerance, cold resistance, and drought resistance of different grape varieties and compare these results with model predictions to assess their adaptability to future climate conditions [61, 74]. At the same time, measured data from ecological sites (such as phenology databases) are used to verify the rationality of the model predictions [62]. Previous research results indicate that certain wild grape species (such as *V. labrusca* and *V. davidii*) possess stronger stress resistance and future climate adaptability, and their desirable genes can be introduced into cultivated varieties through hybrid breeding or gene-editing techniques to enhance the climate adaptability of grapes [32,63,64]; our results are consistent with those previously reported in the literature (Figs 5 and 6). It is also recommended to strengthen the conservation and utilization of wild grape germplasm resources, establish a gene bank, and pay attention to the emerging cultivation potential in high latitude or high altitude areas [60]. In terms of cultivation techniques, promoting water-saving irrigation, scientific application of organic fertilizers, canopy management, and green pest control can effectively enhance the stress resistance and quality of grapes. Governments should increase investment in infrastructure construction in grape-growing areas to improve irrigation, drainage, and transportation conditions and establish a comprehensive policy system for the grape industry, providing technical training and financial support [60,65]. The implementation of these measures can effectively address the potential impacts of climate change on the grape industry, enhance the adaptability and competitiveness of grape production, and provide strong support for the sustainable development of the grape industry.

## Conclusion

Our study employed meteorological factors and the maxent model to predict shifts in suitable agricultural regions for both cultivated and wild grapes under future climatic conditions. The research underscores the enhanced adaptability of North American and East Asian wild grapes in response to projected climate changes. We identified that these specific wild grape species are likely to expand their suitable habitats in future climates, reflecting their sensitivity and adaptability to changing environmental conditions, allowing them to survive and thrive in warmer climates. This insight is significant for understanding the geographical distribution and ecological resilience of grape species. Furthermore, our research provides a foundation for further investigations into resistance gene mining and rootstock improvement (Table. S1), both of which are crucial for addressing climate change challenges and ensuring the sustainable development of the grape industry. In conclusion, our study provides a scientific basis for the strategic planning of grape cultivation regions and offers a novel perspective on the conservation and utilization of grape genetic resources. Future research will focus on genomic analysis of these wild grapes and the application of their resistance genes in the improvement of cultivated varieties.

## Materials and methods

### Data collection and analysis of *Vitis* L. distribution

We comprehensively collected distribution data for *Vitis* L., a process that involved multiple sources to ensure the comprehensiveness and accuracy of the data. First, we obtained distribution data from the Global Biodiversity Information Facility (GBIF) website (https://www.gbif.org/), an international network that aims to provide free and open biodiversity data. Through this platform, we searched for the scientific name of the target species and further limited the search using filters, such as latitude and longitude information, a specific time frame, or a specific region. We are able to access species records and observational data from all over the world, which provide us with a global perspective on the distribution of *Vitis* L. Secondly, we referred to the database of VIVC (Vitis International Variety Catalogue, https://www.vivc.de/), an international catalogue that provides detailed information on grape varieties, including passport data of varieties (such as origin, characteristics), SSR marker data for genetic analysis, literature records, and photos. It not only supplements missing geographic coordinate information to eliminate positioning issues caused by unclear descriptions but also regularly updates to ensure the timeliness and accuracy of the data. In addition to the above two databases, we also referred to relevant literature, especially the study published by Puga *et al.* in 2022 [66], which provided us with data on the distribution of grape species. In addition, Flora of China and Flora of North America are based on scientific plant taxonomy and record information such as morphological characteristics, distribution ranges, and ecological environment of plants. These floras undergo rigorous peer review and field verification to ensure their scientific accuracy. They verify the distribution locations with clear latitude and longitude records using them, supplementing distribution data lacking geographic coordinates and eliminating those that cannot be accurately positioned due to missing or unclear geographic information. Finally, 5662 effective distribution sites were obtained (2109 for cultivated grapes and 3553 for wild grapes). The obtained data, which retain only the longitude, latitude, and species name, are saved in CSV format and used as the source for establishing the species distribution model (SDM) [67, 71].

### Source and processing of climate data

Current and future climate data were derived from the WorldClim database (https://www.worldclim.org/), with a spatial resolution of 2.5 arc-minute grid (geospatial resolution of approximately 5 km), and includes 19 bioclimatic variables (Bio1~Bio19); these variables cover multiple climate elements such as temperature, precipitation, and humidity, providing us with comprehensive climate background information for analyzing and predicting the factors affecting *Vitis* L. distribution. The WorldClim database is presented in the Supplementary Files. The global climate model selected here was the version of European Climate Earth System Model (EC-Earth): EC-Earth3-Veg, which integrates vegetation cover and land surface processes to more accurately simulate the Earth system, including the atmosphere, ocean, sea ice, land surface, and biogeochemical cycles. Due to the uncertainty of future development models, we used two SSPs for the period 2081–2100: SSP245 (Intermediate Development) and SSP585 scenarios (Fossil-Fueled Development). These SSPs represent two distinct trajectories of future human development patterns. The clear differences between them are particularly beneficial for studying the response of grapevines to climate change.

### Model building, optimization, and evaluation

Species distribution models were developed using MaxEnt version 3.4.3 [68], based on processed occurrence data and selected environmental variables. The maxent model is a widely used species distribution modeling tool that is based on the maximum entropy principle and can predict the potential distribution area of species using limited distribution data. In this analysis, we first cleaned and preprocessed the collected species distribution data to ensure the quality and applicability of the data, combined these data with environmental factors, and input them into the maxent model. Specifically, the parameter settings of the initial model are as follows: 25% distribution points are used as the random test percentage in the model, the default maximum number of background points is 10 000, running 10 model replicates, and Jackknife is used to calculate the contribution rate of environmental factors to explore the relationship between the suitable zone and relationship with environment variables. Model construction accuracy is determined by the area under the curve (AUC), with values ranging from 0 to 1. Higher AUC values indicate better model fit, higher construction accuracy, and greater credibility. An AUC value of 0.5–0.6 indicates failure in model construction, 0.6–0.7 indicates poor simulation effect, 0.7–0.8 indicates average simulation effect, 0.8–0.9 indicates good simulation effect, and 0.9–1 indicates excellent simulation effect [69].

### ArcGIS and R for spatial data analysis and visualization

We utilize the Species Distribution Models (SDMtoolbox 2.0) extension in ArcGIS 10.8.2 to analyze areas of expansion and contraction, effectively predicting and addressing the potential impacts of climate change on the geographical distribution of grape populations [11, 70]. SDMtoolbox is a toolkit specifically designed for species distribution model analysis, which provides a range of functions, including data preprocessing, model training, result evaluation, and visualization. In addition, we are conducting an in-depth analysis of the ratios of expansion and contraction areas for grapes under different climate scenarios, aiming to develop more accurate adaptation strategies, and are using RStudio-2024.04.2 + 764 to create bar charts illustrating the percentages.

To enhance the clarity and comprehensibility of the methodological approach, a comprehensive flowchart was developed to visually illustrate the sequential processes of data collection, modeling, and analysis (Fig. S8).

## Supplementary Material

Web_Material_uhaf104

## Data Availability

All data needed to evaluate the conclusions in this paper are presented in the paper and the supplementary information.
