# Supplementary material for: The dynamics of wild Vitis species in response to climate change facilitate the breeding of grapevine and its rootstocks with climate resilience
Source: Hortic Res. 2025 Apr 10;12(7):uhaf104. doi: 10.1093/hr/uhaf104 (PMC12096287; doi:10.1093/hr/uhaf104)
Supplement: Web_Material_uhaf104 [file web_material_uhaf104.zip › FigS1-S8.pdf]

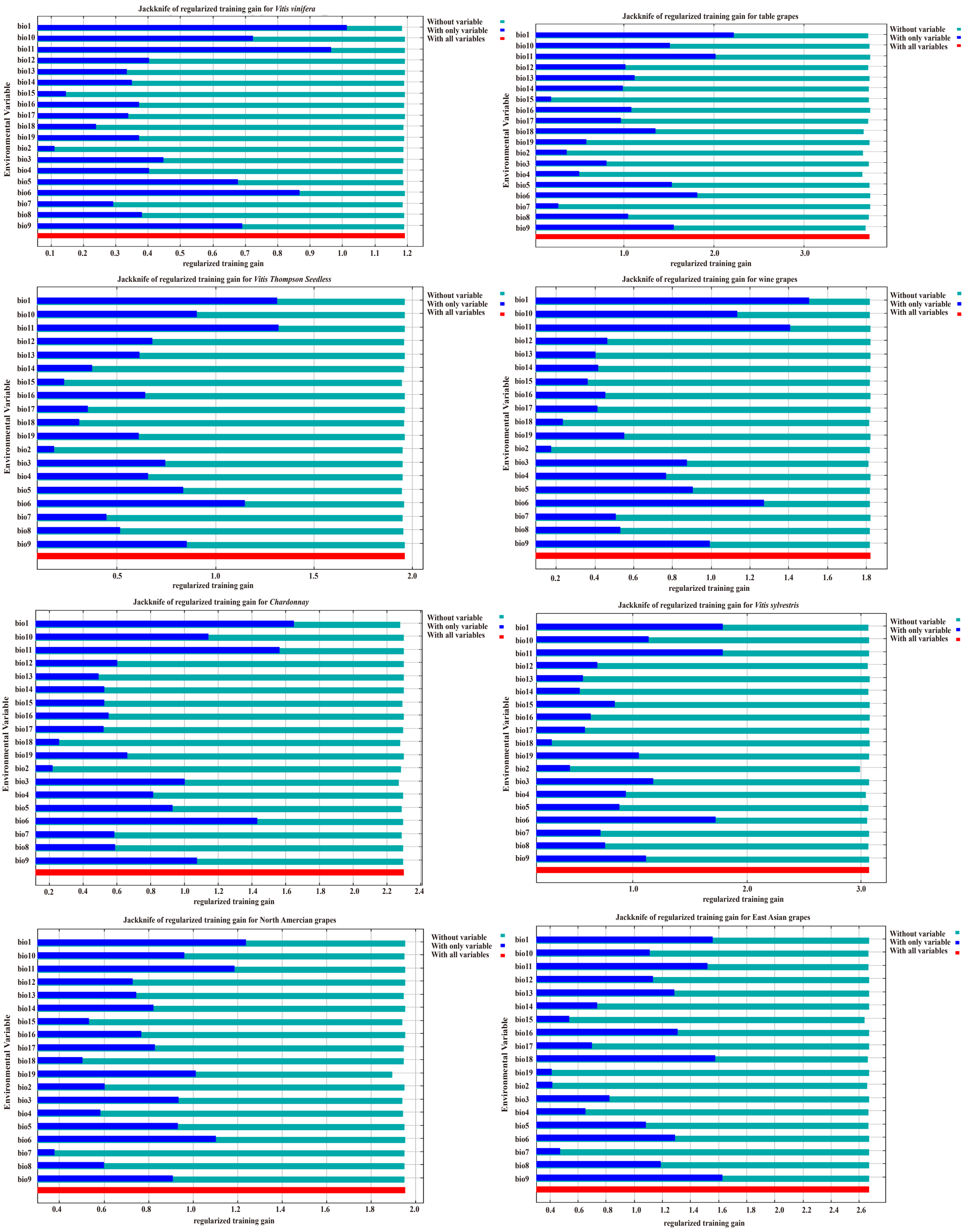

Fig. S1. Evaluation of environmental factors byJakknife method

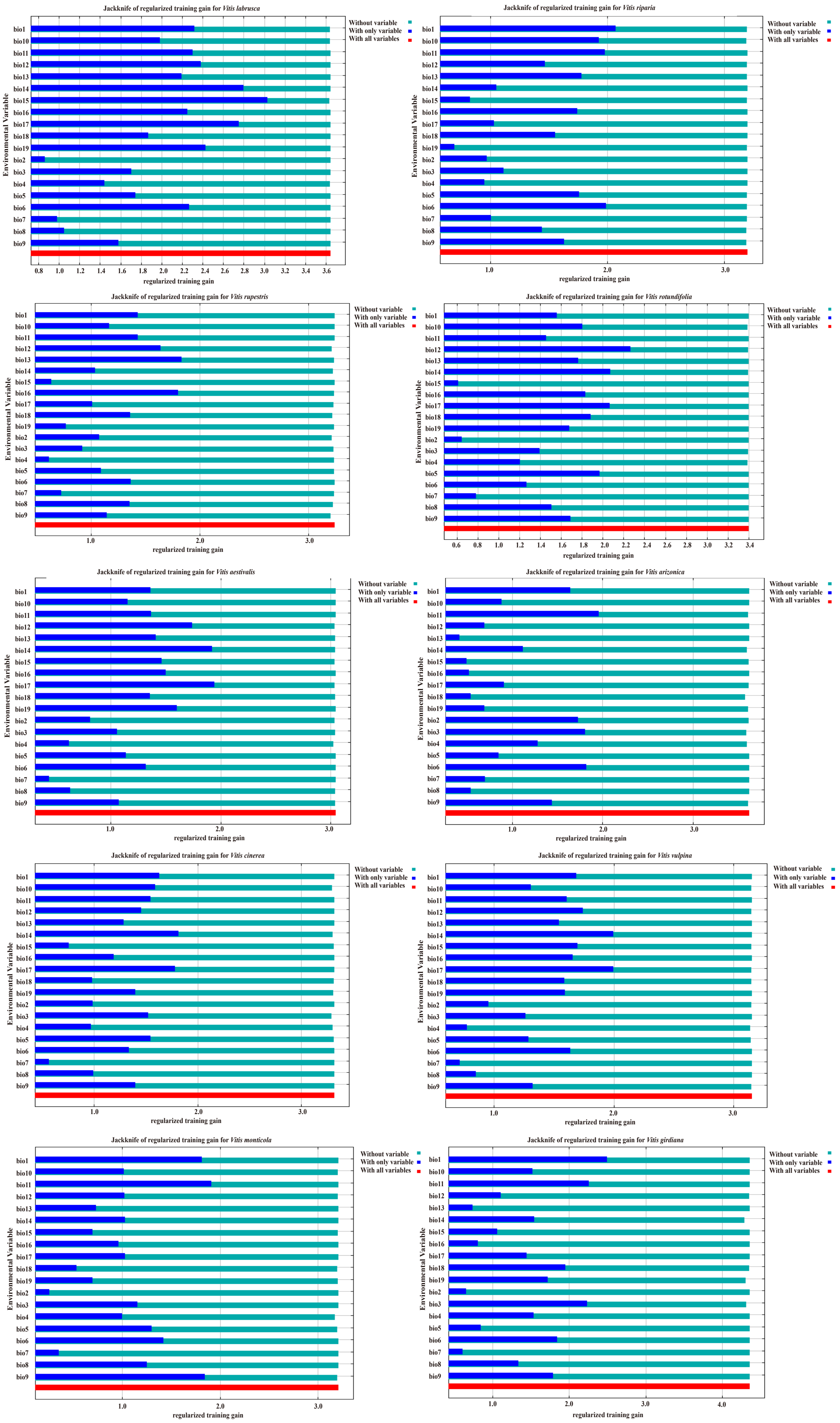

Fig. S1. Evaluation of environmental factors byJakknife method

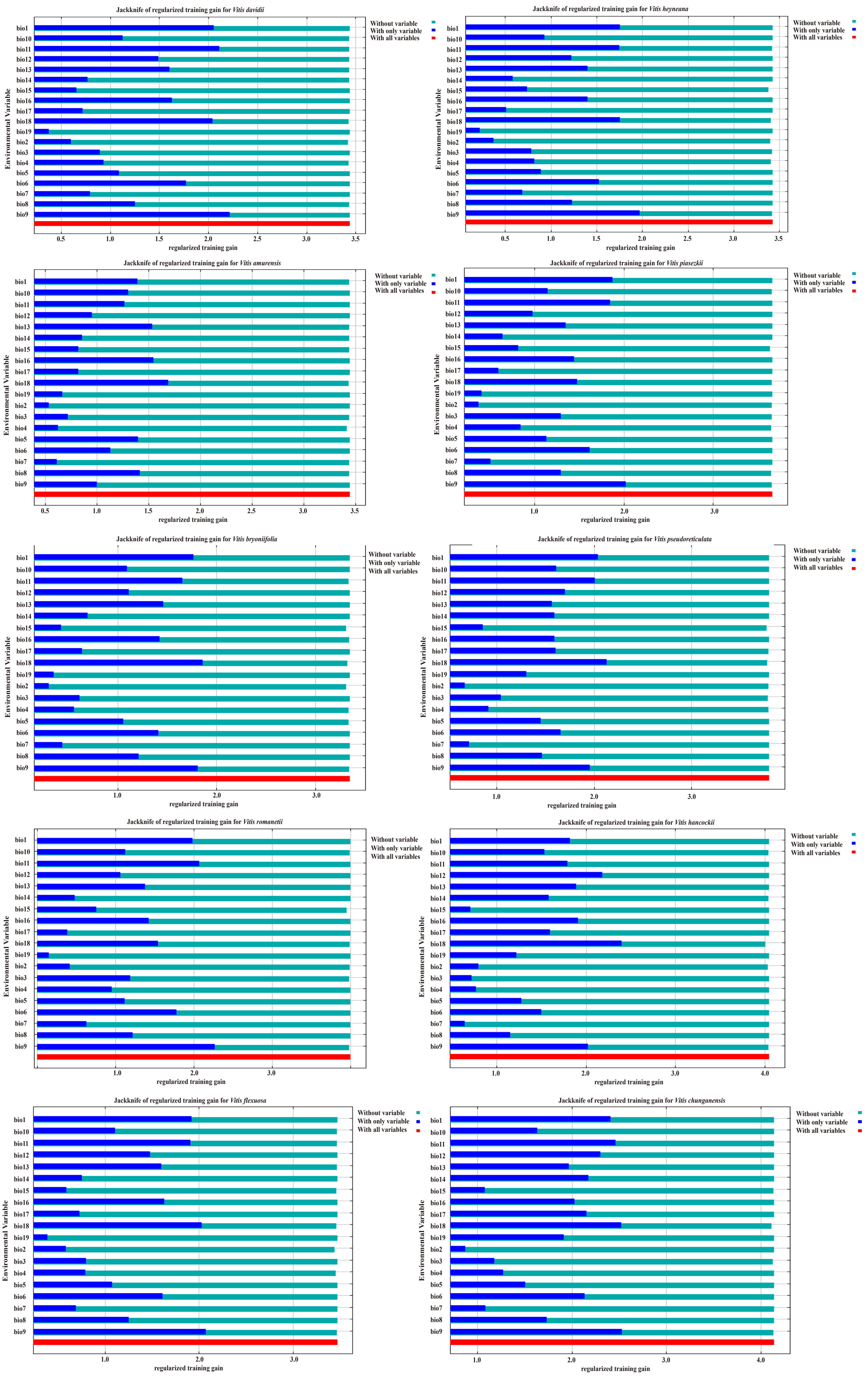

Fig. S1. Evaluation of environmental factors byJakknife method

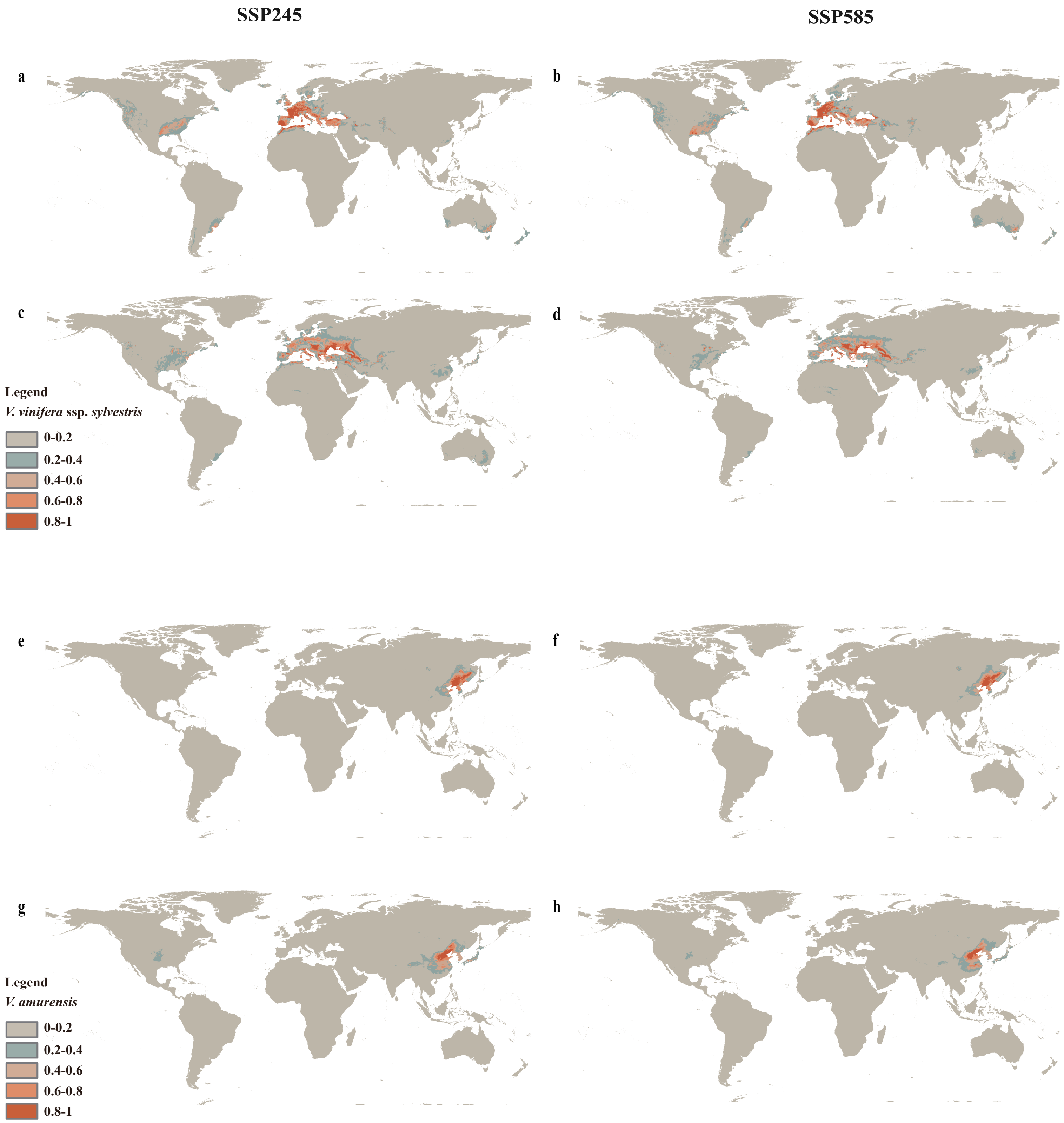

Fig. S2. Maxent model projected bioclimatic suitability for *V. vinifera* ssp. *sylvestris* and *V. amurensis.* (a-b) *V. vinifera* ssp. *sylvestris* east regions, (c-d) *V. vinifera* ssp. *sylvestris* west regions, (e-f) *V. amurensis* northeast regions, (g-h) *V. amurensis* other regions, with future projections for the years 2081–2100 under the SSP245 and SSP585 climatic scenarios.

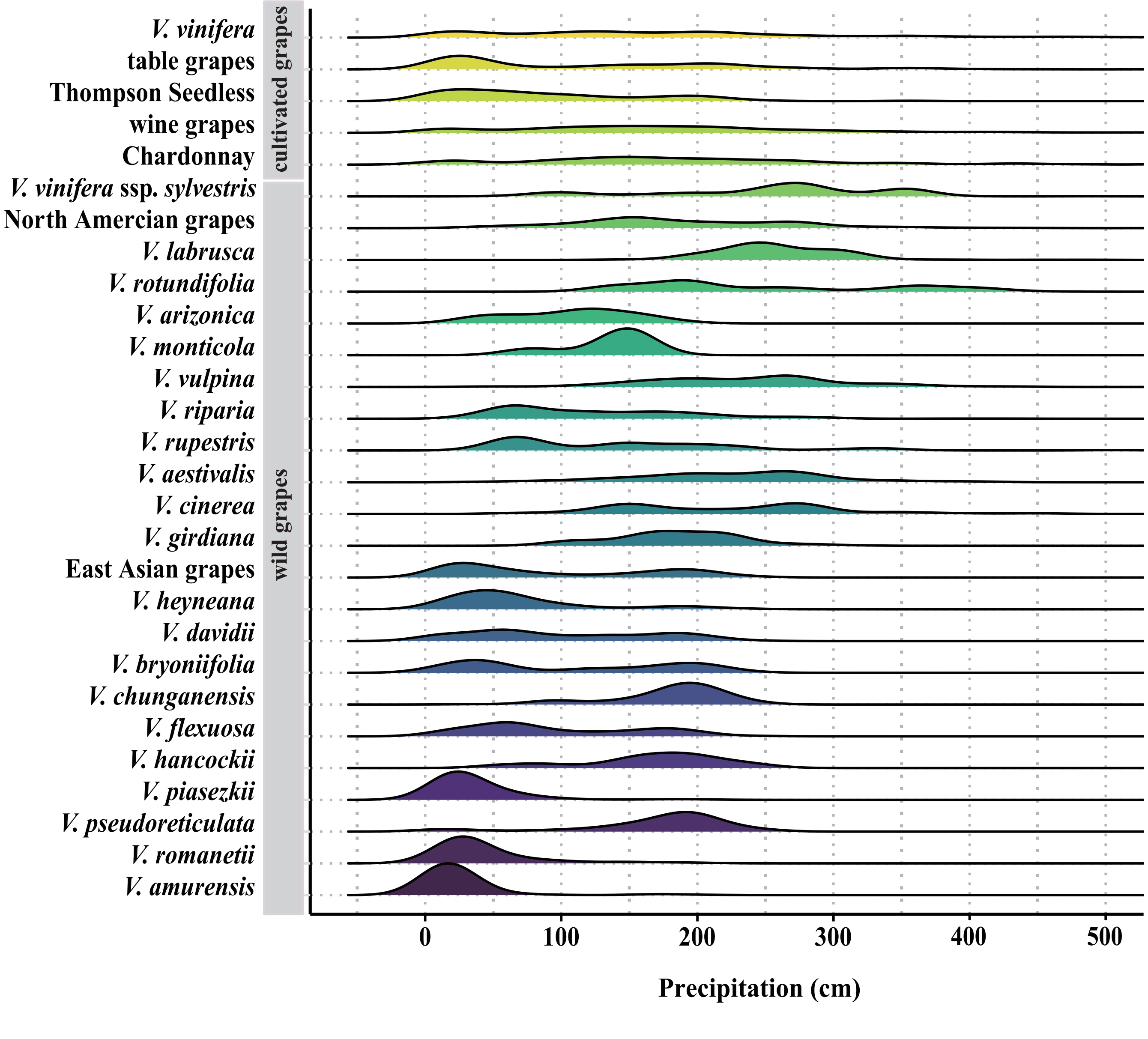

Fig. S3. Precipitation of cultivated and wild grapes: the x-axis distribution indicates plasticity in response to average annual precipitation, while each row on the y-axis distribution represents the names of cultivated grapes and wild grapes, the height of each peak represents the assigned probability.

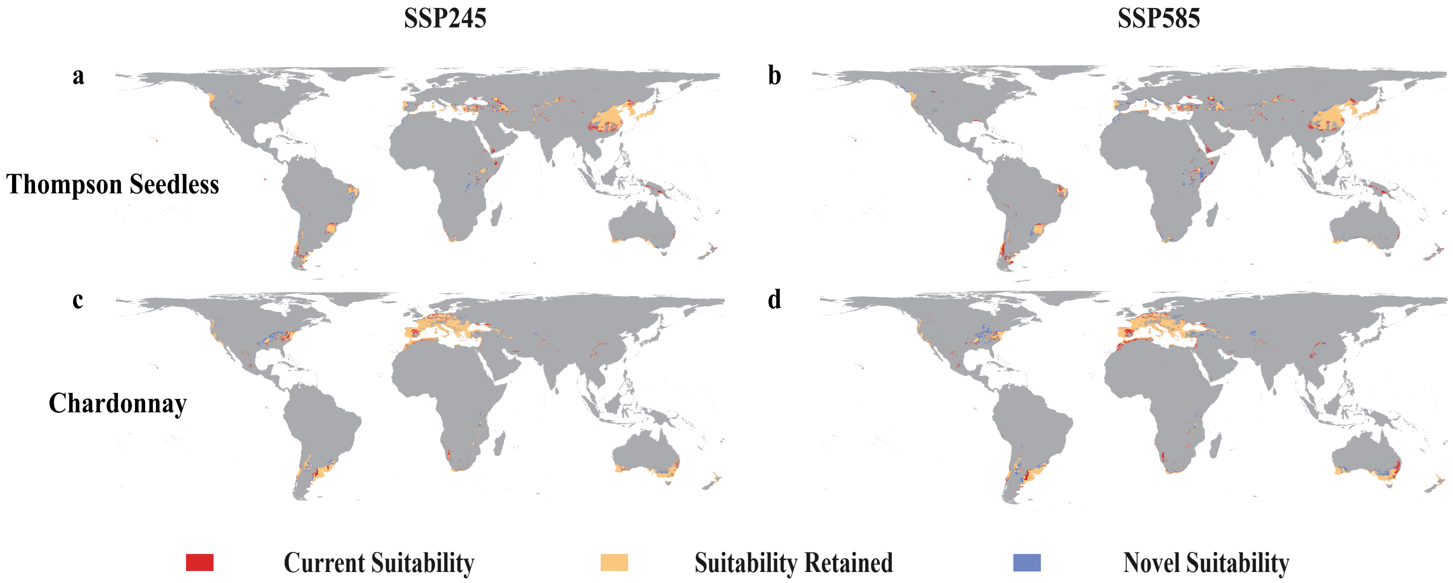

Fig. S4. Maxent model projected bioclimatic suitability for Thompson Seedless and Chardonnay (a-b) Thompson Seedless, (c-d) Chardonnay. Future bioclimatic suitability projections (2081–2100), under the SSP245 and SSP585 climatic scenarios.

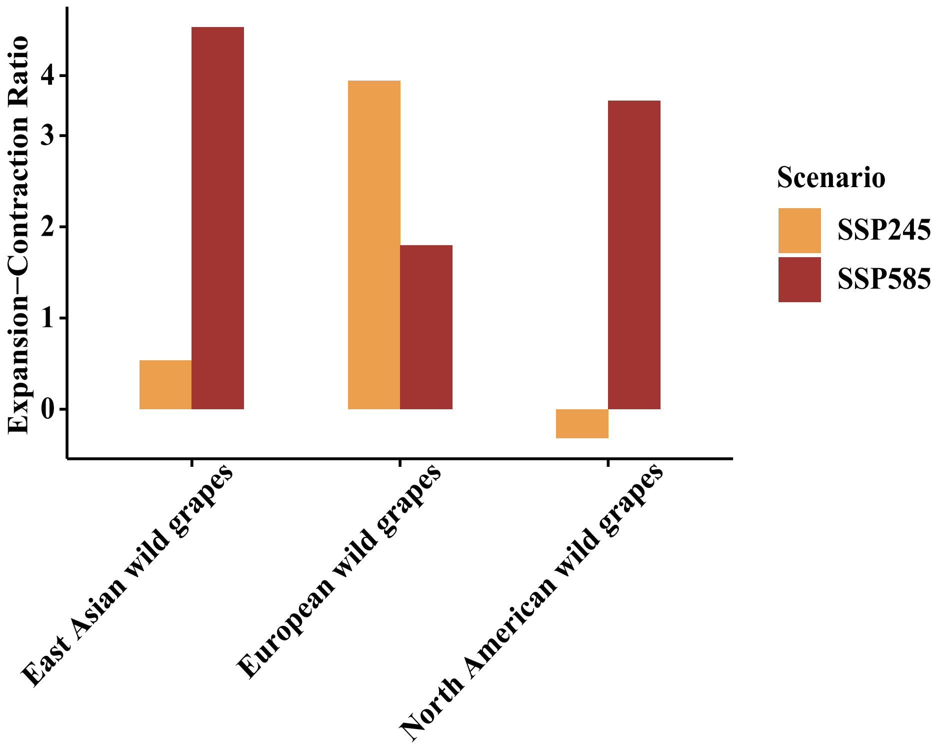

Fig. S5. Net suitability change for the distribution of European wild grapes (*V. vinifera* ssp. *sylvestris*), North American wild grapes, and East Asian wild grapes. Bar plots show Expansion-Contraction Ratio of change in area suitable for grape-range projected by maxent model for SSP245 (yellow) and SSP585 (red).

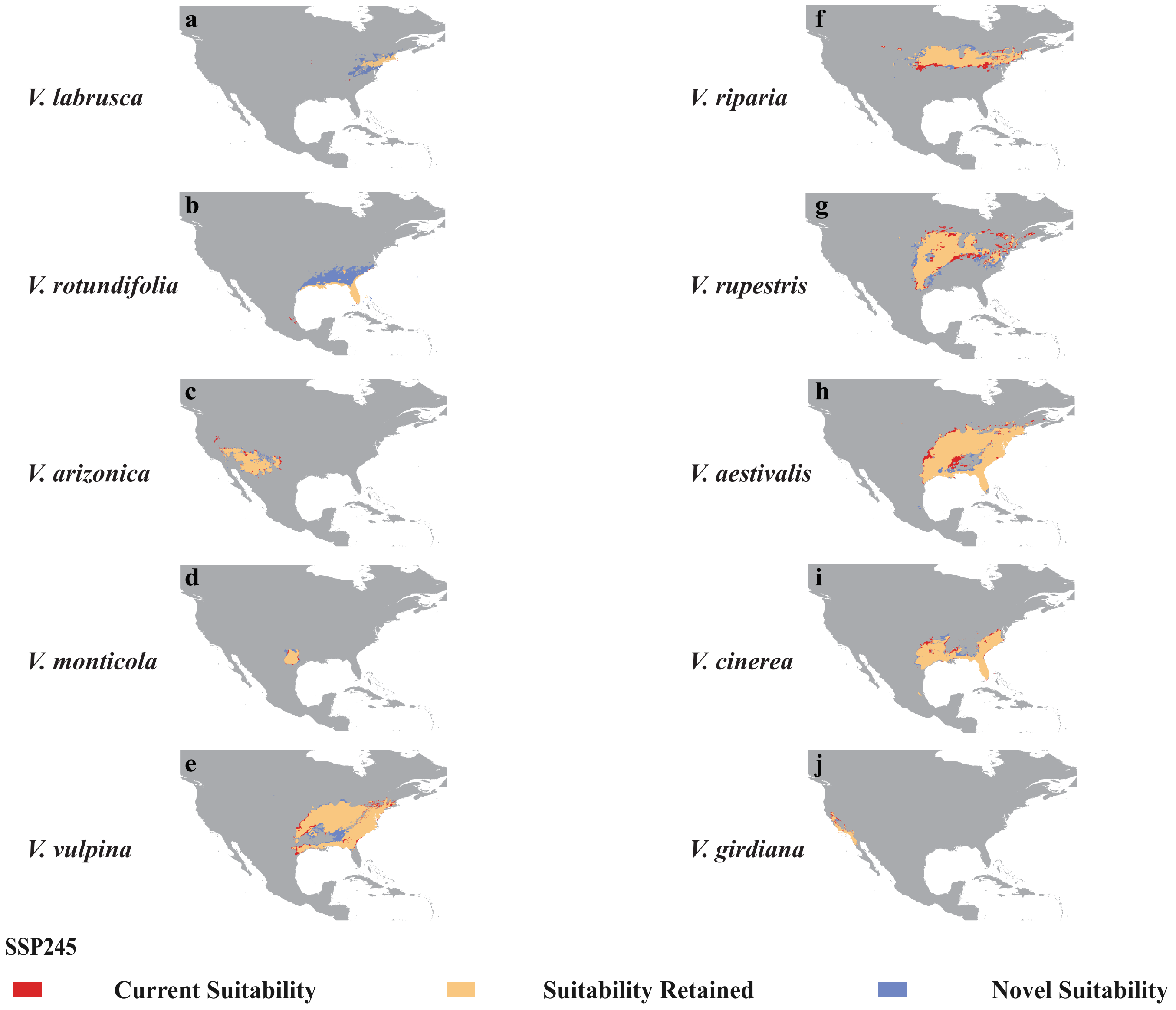

Fig. S6. Maxent model projected bioclimatic suitability for North American wild grapes (a) *V. labrusca*, (b) *V. rotundifolia*, (c) *V. arizonica*, (d) *V. monticola*, (e) *V. vulpina*, (f) *V. riparia*, (g) *V. rupestris*, (h) *V. aestivalis*, (i) *V. cinerea*, (j) *V. girdiana*. Future bioclimatic suitability projections (2081–2100), under the SSP245 climatic scenario.

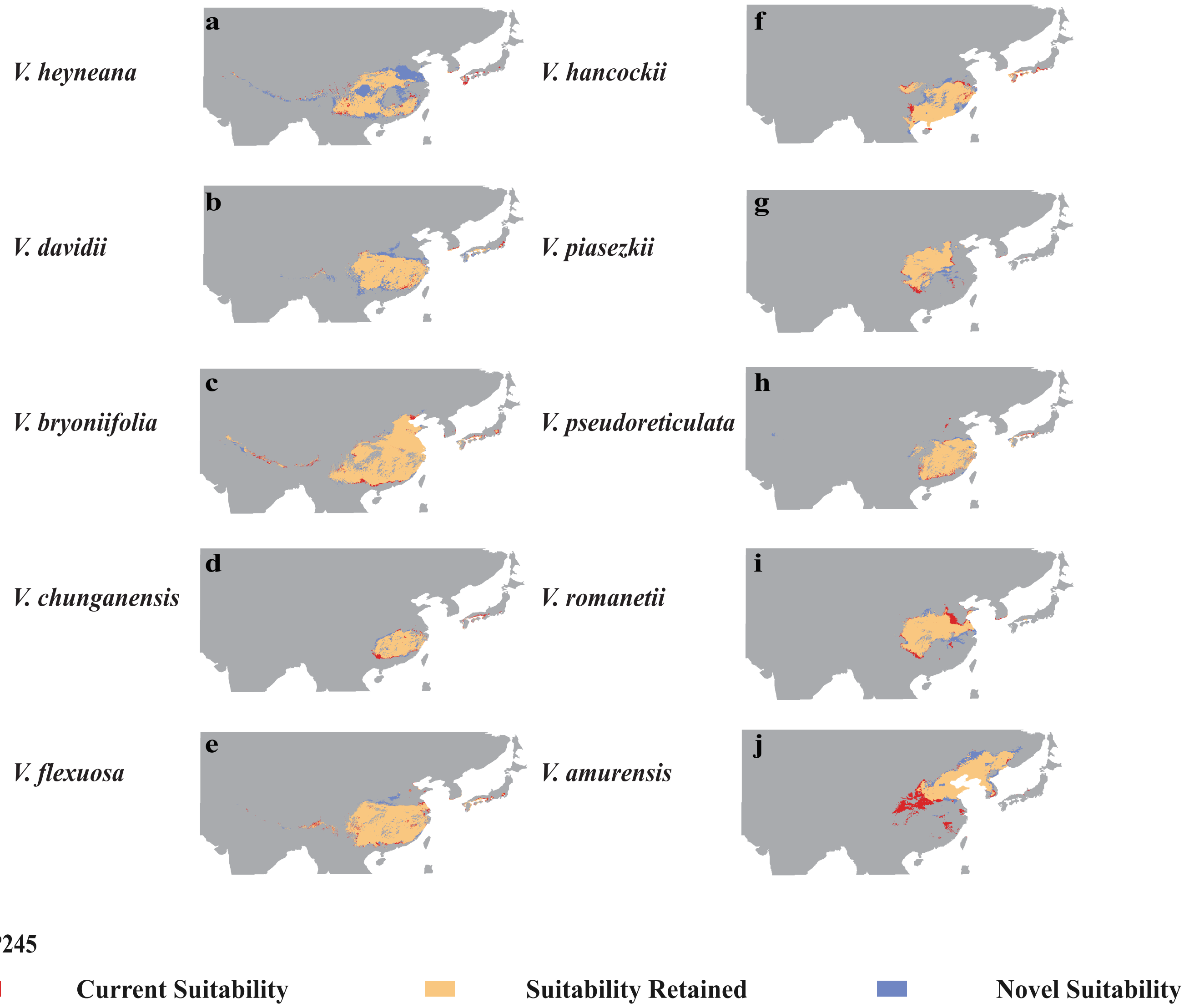

Fig. S7. Maxent model projected bioclimatic suitability for North American wild grapes (a) *V. heyneana*, (b) *V. davidii*, (c) *V. bryoniifolia*, (d) *V. chunganensis*, (e) *V. flexuosa*, (f) *V. hancockii*, (g) *V. piasezkii*, (h) *V. pseudoreticulata*, (i) *V.* romanetii, (j) *V.* amurensis. Future bioclimatic suitability projections (2081–2100), under the SSP245 climatic scenario.

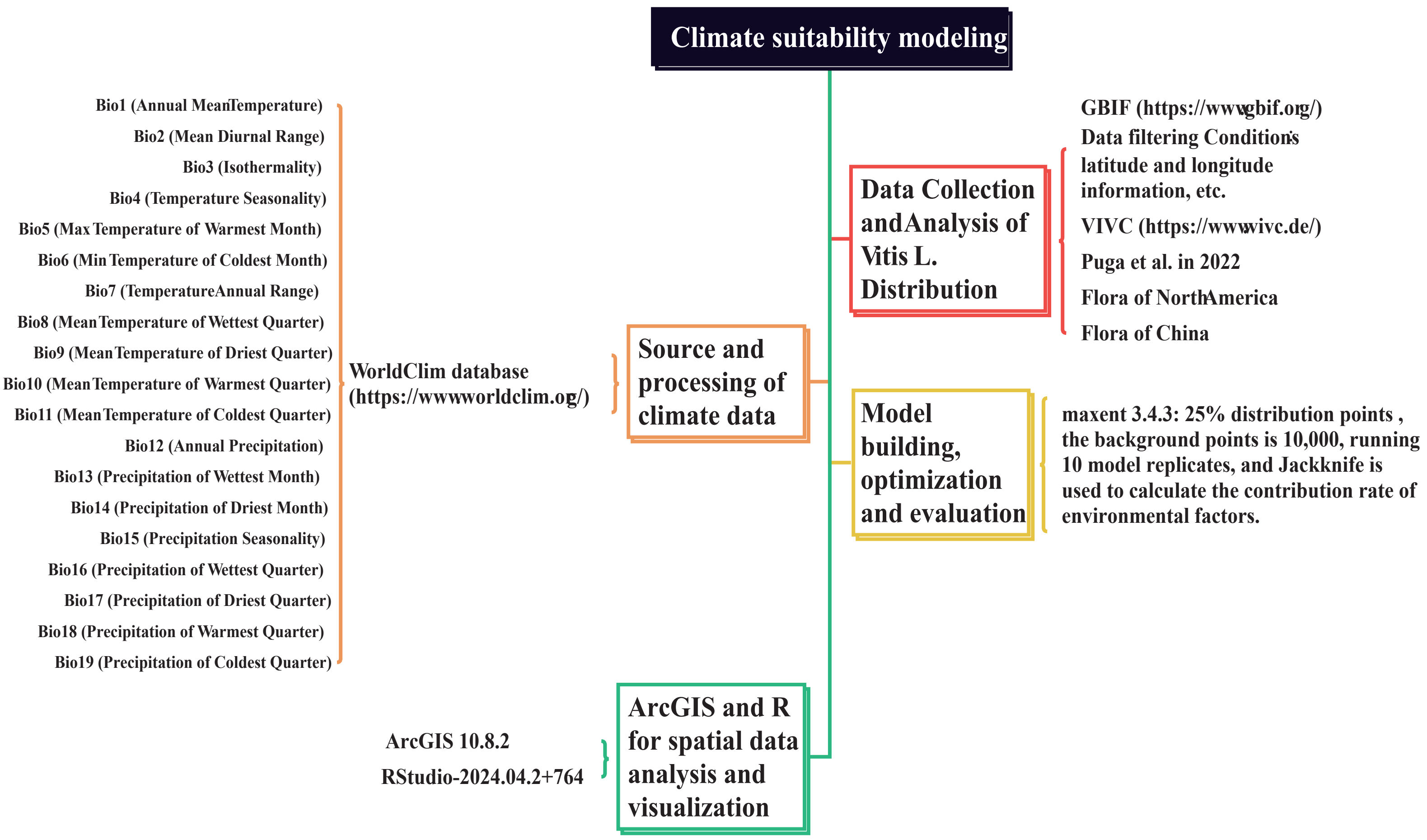

Fig. S8. The process of climate suitability modeling
